# Complete Genome Sequence of a Human Enterovirus 71 Strain Isolated from a Fatal Case in Shanghai, China, in 2012

**DOI:** 10.1128/genomeA.00457-14

**Published:** 2014-05-22

**Authors:** Ying Wang, Qianqian Zhu, Mei Zeng, Ralf Altmeyer, Gang Zou

**Affiliations:** a Unit of Anti-infective Research, Institut Pasteur of Shanghai (International Network of Pasteur Institutes), Chinese Academy of Sciences, Shanghai, China; b Institute of Molecular Ecology and Evolution, Institutes for Advanced Interdisciplinary Research, East China Normal University, Shanghai, China; c Department of Infectious Diseases, Children’s Hospital of Fudan University, Shanghai, China

## Abstract

The complete genome sequence of a human enterovirus 71 strain (SH12-276), isolated from a fatal case in Shanghai in 2012, was determined. Phylogenetic analysis based on the complete genome sequence classified this strain into subgenotype C4.

## GENOME ANNOUNCEMENT

Human enterovirus 71 (EV71), a member of the human enterovirus A species of the family *Picornaviridae*, is one of the main causative agents of hand, foot, and mouth disease (HFMD) in children and infants, predominantly in the Asia-Pacific region (1–4). In particular, EV71 has been associated with more severe cases such as aseptic meningitis, encephalitis, or even death (5–7). Children become susceptible to severe EV71 infections after the loss of maternal antibodies, and one- to two-year-old children are most at risk (8, 9). The genome of EV71 is about 7.4 kb, consisting of a 5′-untranslated region (5′-UTR), P1, P2, P3, and a 3′-untranslated region (3′-UTR) (10). EV71 can be phylogenetically classified into 3 main genogroups (A, B, and C) and 11 genotypes (A, B1 to B5, and C1 to C5) (11). Understanding of the genotypes of EV71 strains in the epidemic regions is important for the development of novel strategies for the prevention and treatment of the diseases associated with EV71.

In this study, a rectal swab was collected from a 6-month-old infant with a clinical diagnosis of hand, foot, and mouth disease at the Children’s Hospital of Fudan University. She had fever, sparse rash on the feet and buttocks, and an oral ulcer for 2 days before admission to the hospital. She developed recurrent vomiting, tachycardia, tachypnea, hypoxemia, and hyperglycemia, and died 3 h after hospitalization. The pathogen was identified as EV71 by means of real-time reverse transcription (RT)-PCR. The clinical isolate was obtained by culturing the clinical sample in RD cells for up to 5 passages, followed by plaque purification. A total of nine sets of primers were designed to amplify the full genome by reverse transcription-PCR, which was available upon request. Sequence of the 5′-end was determined by using the 5′/3′ rapid amplification of cDNA ends (RACE) kit, 2nd generation (Roche), according to the manufacturer’s instructions. The gel-purified RT-PCR products were subject to Sanger sequencing using an ABI 3730xl automatic DNA analyzer. The whole genome of this virus was established by assembling overlapping fragments using the SeqMan program of the Lasergene 7 package (DNASTAR).

The full-length genome of the EV71 strain SH12-276 was composed of 7,405 nucleotides (nt), excluding the poly(A) tail. The 5′-UTR was found to be 742 nt, followed by an open reading frame (ORF) encoding the structural protein P1 (2,586 nt), the nonstructural proteins P2 (1,734 nt) and P3 (2,259 nt), and the 3′-UTR (81 nt). The contents of A, G, C, and U were 27.12%, 23.86%, 23.93%, and 25.09%, respectively, with G+C contents of 47.79%. Phylogenetic trees were constructed by means of the neighbor-joining method with the use of MEGA software, version 5.0, to estimate the viral gene relationship with selected enterovirus strains obtained from GenBank. The results of phylogenetic analyses suggest that SH12-276 belongs to subgenotype C4. Furthermore, SH12-276 was found to be closely related to strain 35/Jingdezhen/China/HFMD_Severe/2011 (GenBank accession no. JQ806378 [98.2% nucleotide identity]) and strain SD09-14/SD/CHN/2009 (GenBank accession no. JX678883 [98.1% nucleotide identity]), which were isolated from different geographic regions in China.

### Nucleotide sequence accession number.

The full-length sequence of SH12-276 isolated in Shanghai in 2012 was deposited in GenBank under the accession no. KC570453.
